# ^18^F-FDG PET/CT based model for predicting malignancy in pulmonary nodules: a meta-analysis

**DOI:** 10.1186/s13019-024-02614-0

**Published:** 2024-03-20

**Authors:** Yu Li, Yi-Bing Shi, Chun-Feng Hu

**Affiliations:** 1 Department of Radiology, Affiliated Hospital of Xuzhou Medical University, Xuzhou, China; 2 Department of Radiology, Xuzhou Central Hospital, Xuzhou, China

**Keywords:** PET/CT, Diagnosis, Pulmonary nodule, Meta-analysis

## Abstract

**Background:**

Several studies to date have reported on the development of positron emission tomography (PET)/computed tomography (CT)-based models intended to effectively distinguish between benign and malignant pulmonary nodules (PNs). This meta-analysis was designed with the goal of clarifying the utility of these PET/CT-based conventional parameter models as diagnostic tools in the context of the differential diagnosis of PNs.

**Methods:**

Relevant studies published through September 2023 were identified by searching the Web of Science, PubMed, and Wanfang databases, after which Stata v 12.0 was used to conduct pooled analyses of the resultant data.

**Results:**

This meta-analysis included a total of 13 retrospective studies that analyzed 1,731 and 693 malignant and benign PNs, respectively. The respective pooled sensitivity, specificity, PLR, and NLR values for the PET/CT-based studies developed in these models were 88% (95%CI: 0.86–0.91), 78% (95%CI: 0.71–0.85), 4.10 (95%CI: 2.98–5.64), and 0.15 (95%CI: 0.12–0.19). Of these endpoints, the pooled analyses of model sensitivity (I^2^ = 69.25%), specificity (I^2^ = 78.44%), PLR (I^2^ = 71.42%), and NLR (I^2^ = 67.18%) were all subject to significant heterogeneity. The overall area under the curve value (AUC) value for these models was 0.91 (95%CI: 0.88–0.93). When differential diagnosis was instead performed based on PET results only, the corresponding pooled sensitivity, specificity, PLR, and NLR values were 92% (95%CI: 0.85–0.96), 51% (95%CI: 0.37–0.66), 1.89 (95%CI: 1.36–2.62), and 0.16 (95%CI: 0.07–0.35), with all four being subject to significant heterogeneity (I^2^ = 88.08%, 82.63%, 80.19%, and 86.38%). The AUC for these pooled analyses was 0.82 (95%CI: 0.79–0.85).

**Conclusions:**

These results suggest that PET/CT-based models may offer diagnostic performance superior to that of PET results alone when distinguishing between benign and malignant PNs.

## Introduction

Pulmonary nodules (PNs) are small (≤ 3 cm) lesions surrounded by lung parenchymal tissue that are not transparent and not the results of atelectasis, mediastinal lymphadenopathy, or pleural effusion [1–3]. In cases where these nodules are > 6 mm in size, computed tomography (CT)-based routine follow-up is warranted [4], with a 1.1-fold increase in the risk of PN malignancy with each 1 mm increase in diameter [5]. Analyses of patient clinical data and CT imaging findings are the most commonly used approach to PN diagnosis [6–8].

CT features often indicative of PN malignancy include CT bronchus sign, vascular convergence sign, pleural retraction, lobulation, and spiculated sign [6–8]. Clinical risk factors for PN malignancy include more advanced age, elevated serum levels of tumor marker proteins, and a history of smoking [6, 9]. Researchers have devised an array of predictive models based on these clinical and imaging features with the goal of more reliably identifying malignant PNs [6–8]. Most CT-derived imaging features, however, are classified as binary variables that can be inconsistently identified based on the experience level of the attending physician. More reliable quantitative imaging strategies are thus needed to minimize this potential for bias, thereby increasing the odds of accurately diagnosing PNs.

^18^F-fludeoxyglucose (^18^F-FDG) positron emission tomography (PET)/CT scans have emerged as a powerful approach to PN diagnosis, with standardized maximum uptake values (SUV_max_) serving as a proxy for radiotracer uptake on imaging scans [10]. Given these advantages, researchers have also incorporated PET/CT imaging parameters into predictive models designed to diagnose PNs in an effort to achieve superior accuracy [11–23]. However, there has been substantial variability among studies with respect to the purported diagnostic performance of these individual PET/CT-based models [11–23]. There thus remains the pressing need for large-scale analyses capable of systematically clarifying the diagnostic utility of the models developed to date.

Accordingly, the present meta-analysis was conducted to clarify the diagnostic performance of PET/CT-based models when used for the differential diagnosis of potentially malignant PNs.

## Materials and methods

### Study selection

Studies of potential relevance were identified by searching the Web of Science, PubMed, and Wanfang databases for all articles published through September 2023 based on the following search strategy: (((((positron emission tomography) OR (PET/CT)) AND (model)) AND ((lung) OR (pulmonary))) AND (nodule)) AND ((((differential) OR (diagnosis)) OR (probability)) OR (predictive)). This meta-analysis was registered at https://inplasy.com/ (No. INPLASY2023100042).

To be eligible for inclusion, studies had to be: (1) focused on the differential diagnosis of malignant or benign PNs, (2) centered on the development or testing of PET/CT-based models that were provided within the study, and (3) transparent with respect to the true positive (TP), true negative (TN), false positive (FP), and false negative (FN) values associated with the tested models. provided. Case reports, non-human studies, and reviews were excluded from this study.

### Data extraction and quality analyses

Two investigators were responsible for independently extracting pertinent data from these studies, including baseline study data, baseline patient data, and the results of diagnostic analyses. Any discrepancies were resolved by a third investigator. The QUADAS-2 tool was used to gauge risk of bias [24].

### Definitions

TP results were those for which both PET/CT-based models and final diagnoses were indicative of PN malignancy, whereas FP results were those for which PET/CT-based models predicted that a given lesion was malignant but it was ultimately found to be benign. Conversely, TN results were those for which both PET/CT-based models and final diagnoses indicated that a PN was benign, whereas FN results were those for which PET/CT-based models predicted that a given lesion was benign but it was ultimately found to be malignant.

### Meta-analysis

Stata v 12.0 (Stata Corporation, TX, USA) was used to compute pooled sensitivity, specificity, diagnostic score, negative likelihood ratio (NLR), positive likelihood ratio (PLR), and summary receiver operating characteristic (SROC) curves for this study. A given predictive model was considered to exhibit high diagnostic performance if it exhibited an NLR < 0.2 or a PLR > 5. An area under the SROC curve (AUC) value greater than 0.8 was also considered to indicate a high degree of diagnostic utility [3]. RevMan v 5.3 was used to compare pooled SUV_max_ values between benign and malignant PNs. I^2^ values were employed to gauge the degree of heterogeneity, with I^2^ > 50% indicating that such heterogeneity was significant. The possibility of publication bias was assessed with Deeks’ funnel plots, and *P* < 0.05 served as the threshold for defining statistical significance.

## Results

### Study selection

**Fig. 1:**
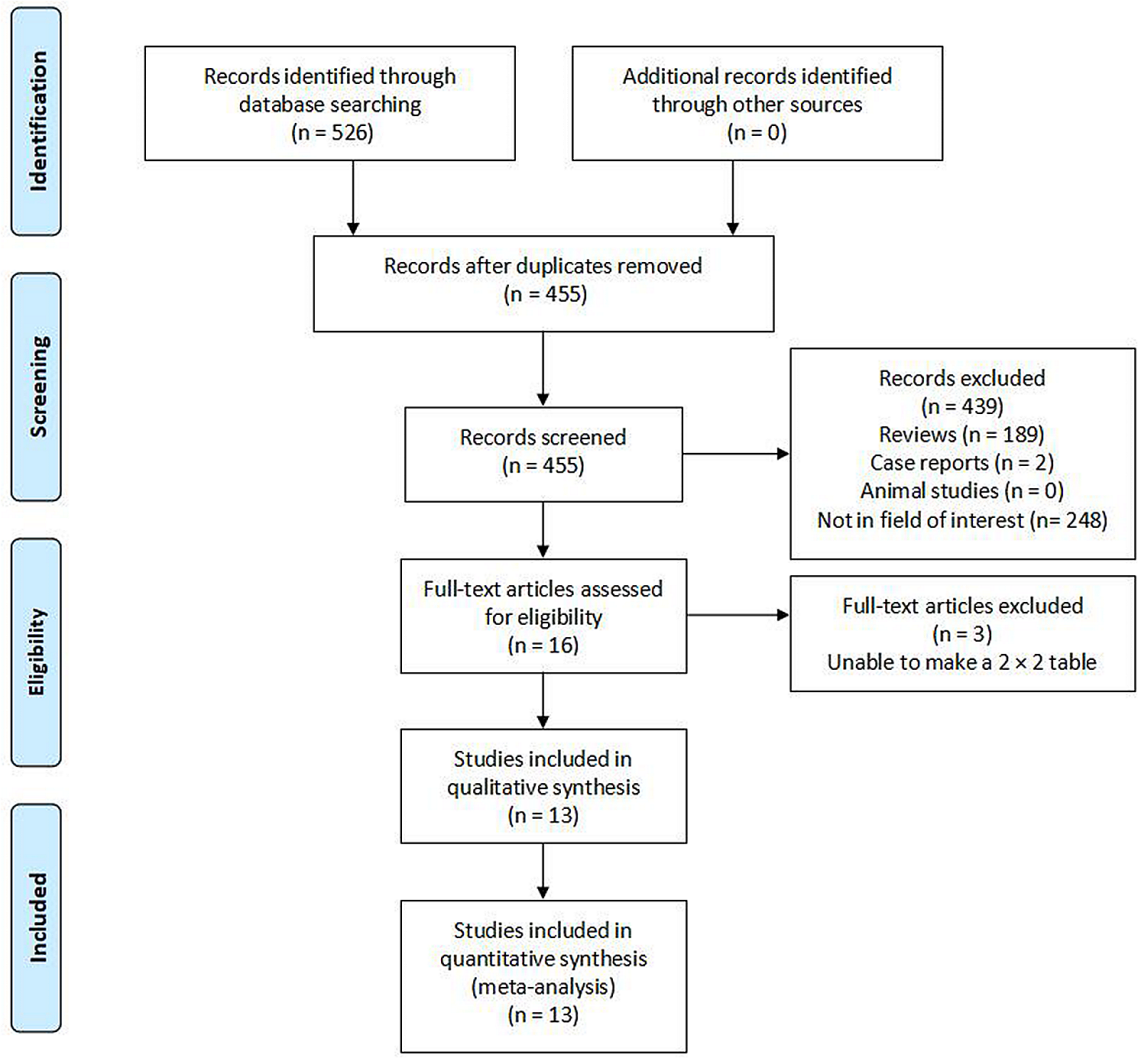
The study selection process for this meta-analysis

The initial search strategy returned 526 studies of which 13 were found to be relevant and incorporated into the final analyses (Fig. 1). These 13 studies were retrospective in design, and included 11 and 2 studies respectively conducted in China and Spain. For further study-specific details, see Table 1.

**Table 1:** Characteristics of studies included in meta-analysis

Studies	Year	Country	Blind	Sample size	Male/Female	Age (y)	Malignant/Benign	Reference standard
Chen [11]	2013	China	Unclear	109	55/54	24–80	67/42	S, B
Cheng [12]	2019	China	Unclear	362	194/168	22–68	291/71	S, B
Guo [13]	2019	China	Unclear	312	172/140	30–89	215/97	S, B
Honguero Martínez [14]	2021	Spain	Unclear	305	225/80	29–86	258/47	S, B
Lin [15]	2015	China	Yes	186	Not given	Not given	123/63	S, B
Liu [16]	2016	China	Unclear	164	103/61	Mean 58	104/60	S, B, F
Ma [17]	2020	China	Unclear	161	91/70	27–85	131/30	S, B
Pei [18]	2015	China	Unclear	156	92/64	Mean 57.6	85/71	S, B
Tian [19]	2012	China	Unclear	105	71/34	Mean 57	61/44	S, B
van Gómez López [20]	2015	Spain	Unclear	55	45/10	Mean 61	40/15	S
Wang [21]	2018	China	Yes	177	95/82	26–85	119/58	S, B
Xiang [22]	2016	China	Yes	110	71/39	Mean 59	80/30	S
Zhang [23]	2021	China	Unclear	222	All males	Mean 68	157/65	S, B, F

B: biopsy; F: follow-up; S: surgery

A total of 1,731 and 693 malignant and benign PNs were ultimately included in these studies. Numbers of predictors included in individual predictive models ranged from 2 to 7 (Table 2). Except for PET/CT, age was the predictor in 12 of the 13 models. The common malignant CT features, such as lobulation, spiculation, and pleural retraction, occurred in 6, 5, and 3 models. Different models could provide different performances and therefore a different number of TP, TN, FP, FN. For details regarding raw TP, FP, TN, and FN data, see Table 3.

**Table 2:** The details of each predictive model

	Number of factors	Items of predictive factors
Chen [11]	7	Age, density, lesion-lung border, lobulation, concentrated vessel, pleural retraction, PET
Cheng [12]	6	Age, vacuole, lobulation, calcification, diameter, PET
Guo [13]	7	Age, diameter, smoking history, spiculation, lobulation, cavity, PET
Honguero Martínez [14]	4	Age, sex, malignant history, PET
Lin [15]	5	Age, lobulation, concentrated vessel, pleural retraction, PET
Liu [16]	3	Age, spiculation, PET
Ma [17]	4	Age, concentrated vessel, calcification, PET
Pei [18]	7	Age, sex, size, spiculation, PET, border, concentrated vessel
Tian [19]	6	Age, smoking, gender, diameter, PET, spiculation
van Gómez López [20]	2	Age, PET
Wang [21]	5	Age, lobulation, concentrated vessel, pleural retraction, PET
Xiang [22]	5	Age, PET, lobulation, calcification, spiculation
Zhang [23]	3	Calcification, concentrated vessel, PET

PET: positron emission tomography

**Table 3:** Raw Data of diagnostic performance of studies included in this meta-analysis

	Predictive model				PET alone			
	TP	FP	FN	TP	TP	FP	FN	TP
Chen [11]	168	17	49	67	-	-	-	-
Cheng [12]	259	12	32	56	-	-	-	-
Guo [13]	165	22	50	75	-	-	-	-
Honguero Martínez [14]	235	23	23	24	244	36	14	11
Lin [15]	108	12	15	51	-	-	-	-
Liu [16]	95	12	9	48	98	21	6	39
Ma [17]	108	4	23	26	129	7	2	23
Pei [18]	79	3	6	68	-	-	-	-
Tian [19]	55	7	6	37	-	-	-	-
van Gómez López [20]	35	8	5	7	32	7	8	8
Wang [21]	106	20	13	38	103	33	16	25
Xiang [22]	69	6	11	24	201	38	38	36
Zhang [23]	138	9	19	56	-	-	-	-

FN: false negative; FP: false positive; PET/CT: positron emission tomography/computed tomography; TN: true negative; TP: true positive

### Bias analyses

**Fig. 2:**
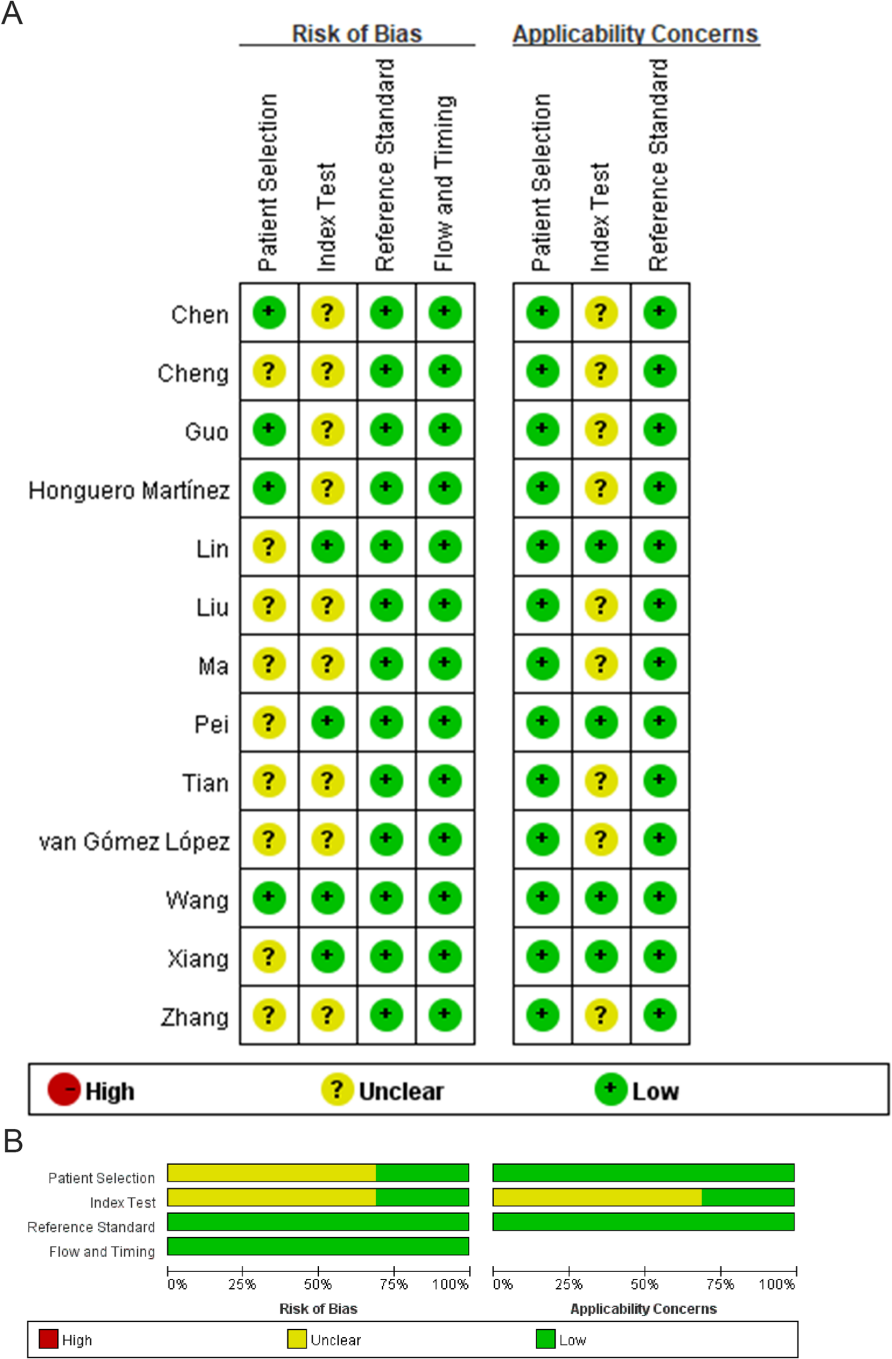
(**A**) The quality assessment of each included study. (**B**) The summary of the quality assessment

The potential for bias was examined using the QUADAS-2 tool (Fig. 2). This approach revealed that 9 of the 13 studies did not indicate whether patients were consecutively enrolled [12, 15–20, 22, 23], and a partially overlapping set of 9 studies did not provide sufficient clarity regarding blinding status [11–14, 16–20]. Reference standards were used for diagnostic confirmation in all studies.

### PET/CT-based model diagnostic performance

**Fig. 3:**
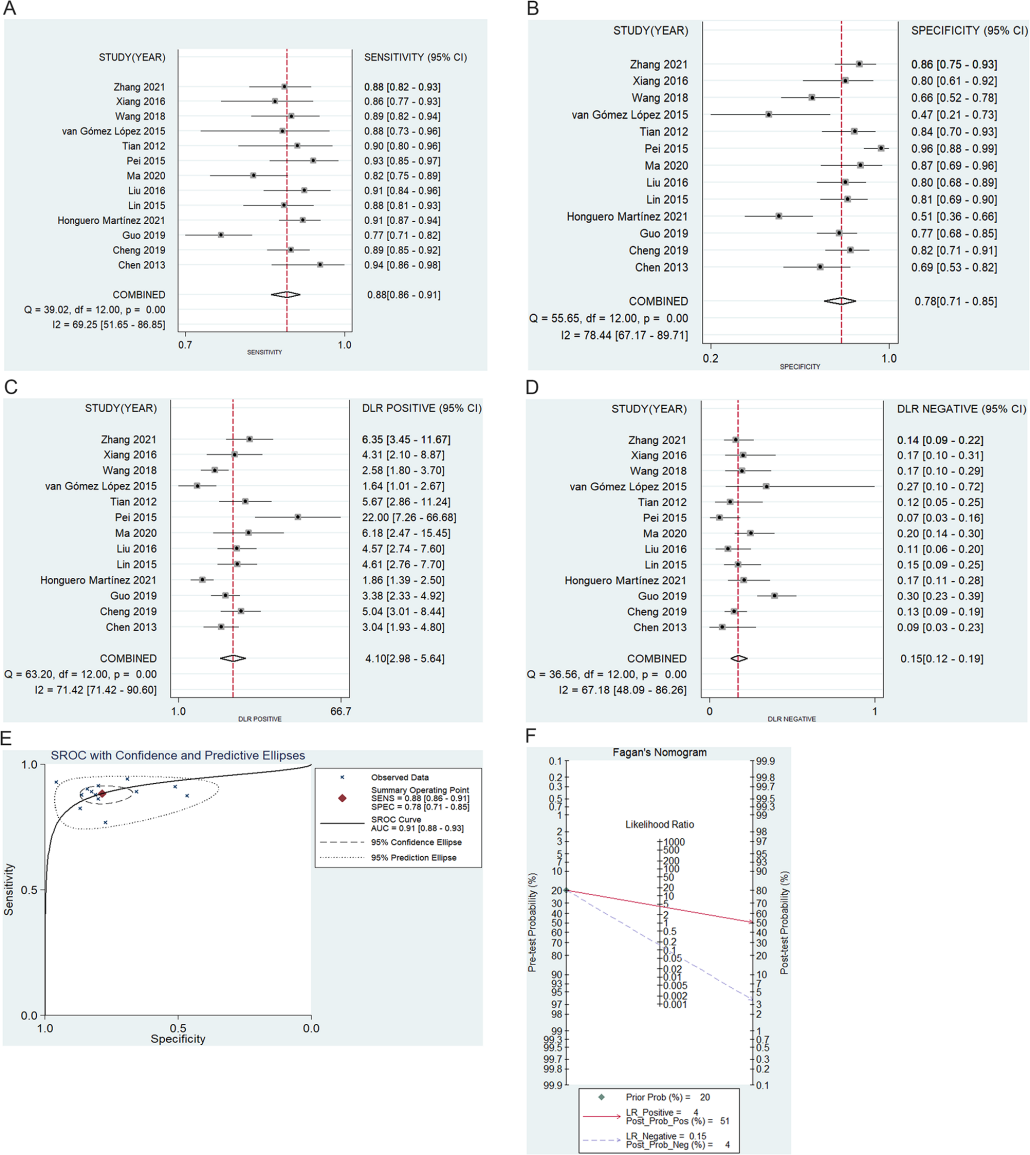
The results of (**A**) sensitivity, (**B**) specificity, (**C**) PLR, (**D**) NLR, (**E**) SROC, and (**F**) Fagan diagram for PET/CT based model

TP, FP, TN, and FN data for PET/CT-based models were provided in all 13 studies. The respective pooled sensitivity, specificity, PLR, and NLR values for these models were 88% (95%CI: 0.86–0.91, Fig. 3a), 78% (95%CI: 0.71–0.85, Fig. 3b), 4.10 (95%CI: 2.98–5.64, Fig. 3c), and 0.15 (95%CI: 0.12–0.19, Fig. 3d), with all four being subject to significant heterogeneity (I^2^ = 69.25%, 78.44%, 71.42%, and 67.18% respectively). The AUC value was 0.91 (95%CI: 0.88–0.93, Fig. 3e), and the SROC curve deviated substantially from a shoulder-like appearance, indicating that a threshold effect is unlikely to influence these results. A Fagan plot with a 20% pre-test probability exhibited respective 51% and 4% post-test PLR and NLR probabilities (Fig. 3f), with no evidence of significant publication bias (*P* = 0.996).

### The diagnostic utility of PET results alone

**Fig. 4:**
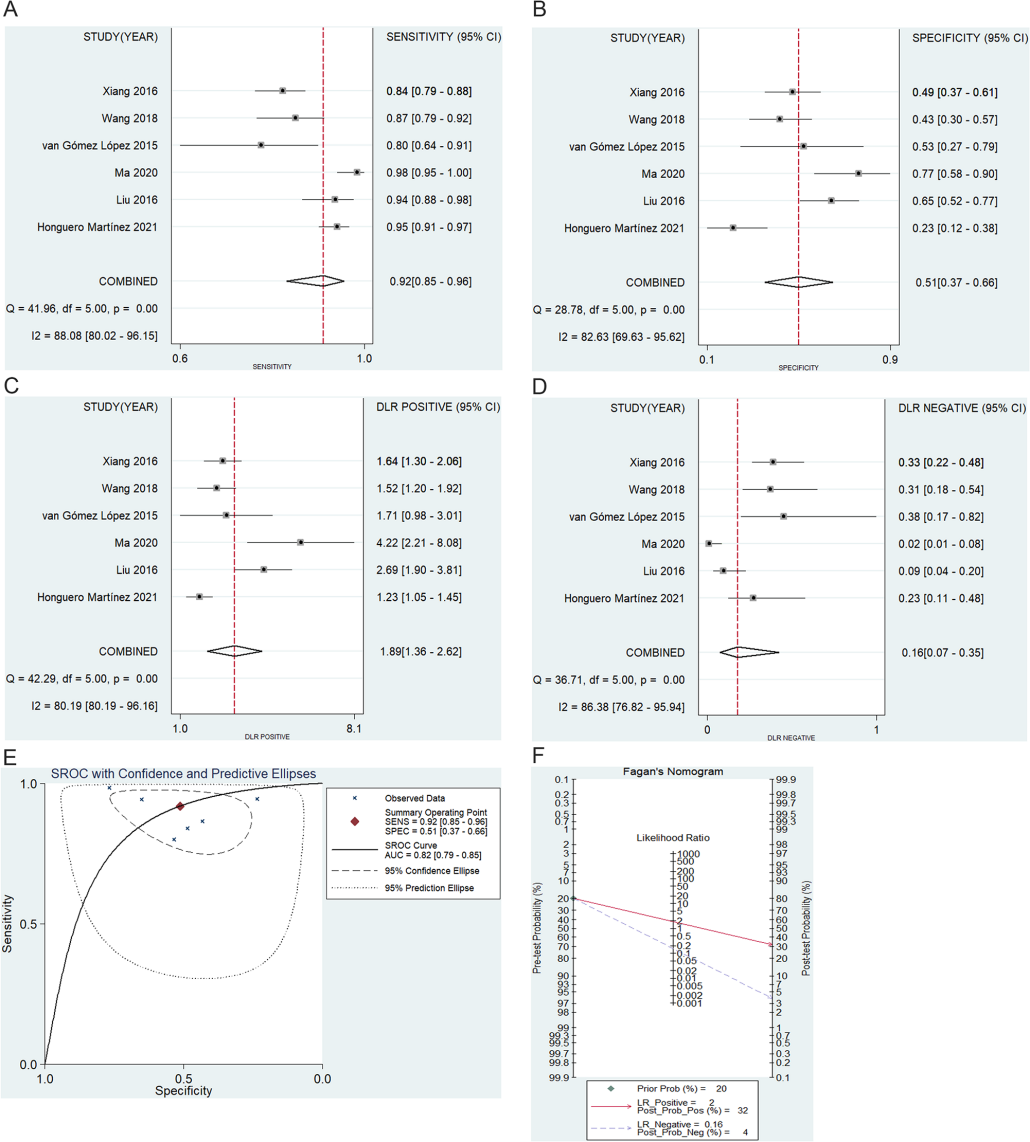
The results of (**A**) sensitivity, (**B**) specificity, **C**) PLR, (**D**) NLR, (**E**) SROC, and (**F**) Fagan diagram for PET/CT alone

Raw TP, FP, TN, and PN data for diagnoses made solely based on PET-derived SUV_max_ values were provided by 6 studies [14, 16, 17, 20–22]. The respective pooled sensitivity, specificity, PLR, and NLR values for diagnoses made based only on these values were 92% (95%CI: 0.85–0.96, Fig. 4a), 51% (95%CI: 0.37–0.66, Fig. 4b), 1.89 (95%CI: 1.36–2.62, Fig. 4c), and 0.16 (95%CI: 0.07–0.35, Fig. 4d), with all four values again being subject to significant heterogeneity (I^2^ = 88.08%, 82.63%, 80.19%, and 86.38%). The corresponding AUC value was 0.82 (95%CI: 0.79–0.85, Fig. 4e), and the appearance of the SROC curve did not reveal any evidence of a threshold effect. A Fagan plot with a 20% pre-test probability exhibited respective 32% and 4% post-test PLR and NLR probabilities (Fig. 4f), with no evidence of significant publication bias (*P* = 0.566).

### SUV_max_ values

**Fig. 5:**
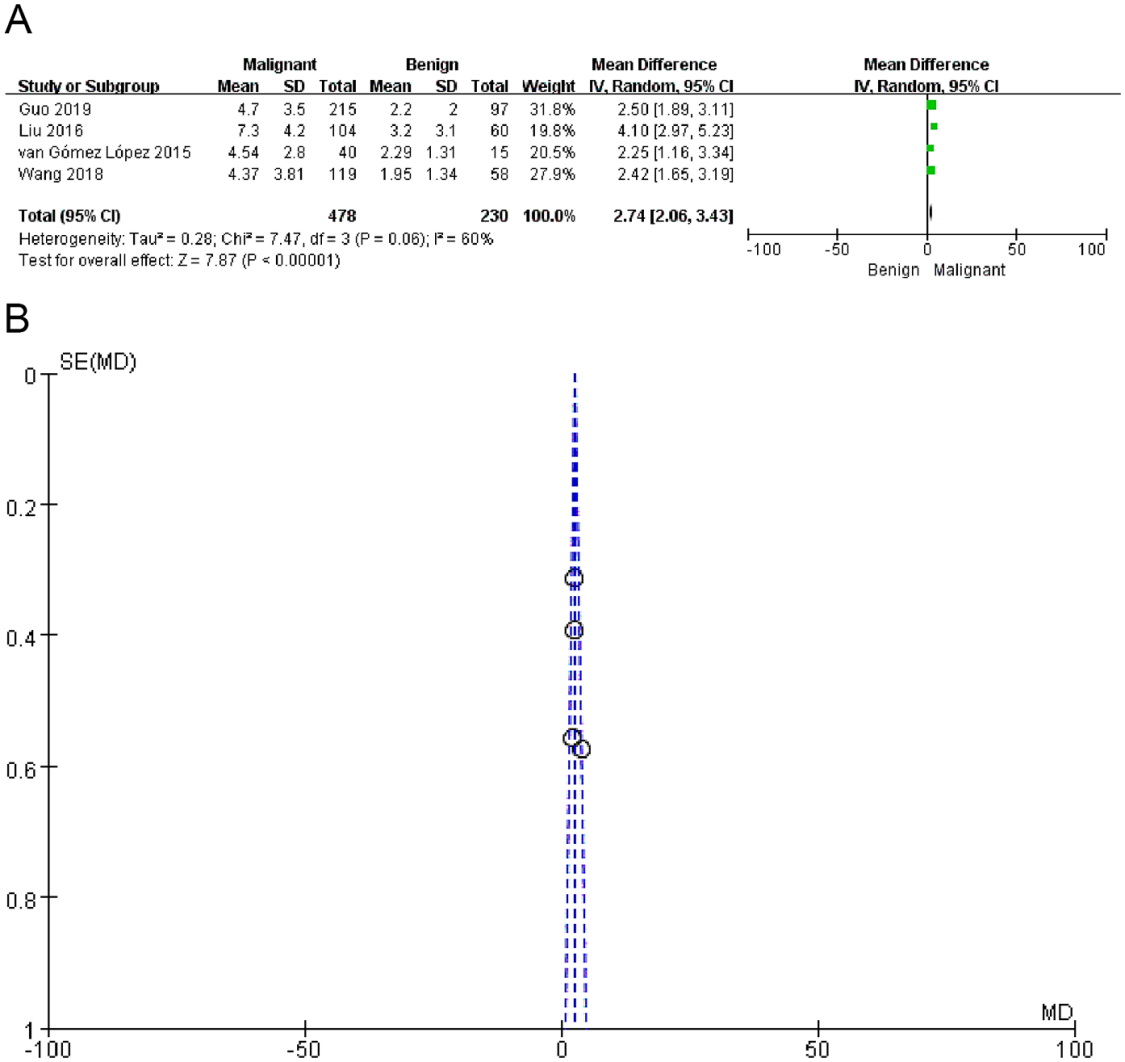
(**a**) The forest plot of the pooled SUV_max_ values between malignant and benign PNs. (**b**) The assessment of the publication bias of SUVmax values

The mean SUV_max_ values for benign and malignant PNs were reported in 4 total studies [13, 15, 20, 21]. Significantly higher pooled SUV_max_ values were observed for malignant PNs as compared to benign nodules (*P* < 0.00001, Fig. 5a), although significant heterogeneity was detected (I^2^ = 60%). Sensitivity analyses suggested that the study conducted by Liu et al. [16] was the greatest source of heterogeneity, but even with the removal of this study the pooled SUV_max_ of malignant PNs remained higher than that of benign PNs (*P* < 0.00001). Funnel plots revealed a low risk of publication bias (Fig. 5b).

## Discussion

The present meta-analysis explored the performance of PET/CT-based models as tools for the differential diagnosis of PNs. The overall pooled AUC value of 0.91 was indicative of excellent predictive performance in this context, while the low NLR value (0.15) demonstrates that these PET/CT-based models can satisfactorily diagnose benign PNs when predictive scores fall below the established cut-off value. As the pooled PLR value of 4.10 was less than 5, however, this suggests that the diagnostic ability of these PET/CT-based models for malignant PNs is only moderate when predictive scores fall above the established cut-off value.

PET/CT imaging can yield both CT images that offer morphological insight regarding a given lesion, as well as PET images capable of quantifying glucose metabolism rates. PET scans thus enable the detection of malignant lesions composed of highly metabolically active cells, given that they take up ^18^F-FDG and glucose at higher rates than do benign cells [25, 26]. In the present meta-analysis, a significantly higher pooled SUV_max_ value was exhibited by malignant PNs as compared to benign PNs.

The diagnostic utility of individual CT features is relatively limited when evaluating PNs. In prior meta-analyses assessing the diagnostic performance of lobulation sign, calcification, and spiculation as approaches to differential diagnosis of PNs, the AUC values were between 0.65 and 0.76 [1–3]. The AUC for the diagnostic utility of PET alone in the present study was 0.82, but the pooled specificity was just 51%. High levels of ^18^F-FDG uptake can also be observed for benign inflammatory, infectious, or granulatomous disease-associated lesions [27], contributing to a relatively low PLR of 1.89. The comparison of diagnostic performance between the predictive model and PET alone suggests that the diagnostic ability of PET alone is limited when evaluating PNs, emphasizing the need to combine multiple signs in an effort to improve the performance of diagnostic models.

There are many advantages to utilizing mathematical models when diagnosing PNs. Notably, these models can ensure that patients can be assessed in a more objective manner, yielding a predictive score reflective of the odds of PN malignancy. In addition, these models can provide risk coefficients for all predictive factors incorporated therein, allowing researchers to directly establish the relative risk associated with each incorporated factor.

The Mayo model was the first predictive model designed to distinguish between benign and malignant PNs [28]. Herder et al. [29] combined the Mayo model with PET results to establish the first PET/CT-based model, which exhibited an AUC of 0.92 in line with the pooled AUC measured in the present meta-analysis. This AUC value was also higher than that of the Mayo model (0.79) or PET scanning results alone (0.88) [29].

In addition to imaging features, predictive models can also incorporate levels of tumor markers or particular clinical features [3]. More advanced age and higher serum concentrations of carcinoembryonic antigen have both been linked to a greater risk of PN malignancy [3, 9]. While age was a factor that was included in most predictive analyses analyzed herein, none incorporated tumor markers. Additional research focused on developing new PET/CT-based predictive models incorporating clinical characteristics, imaging features, and tumor marker levels are thus warranted to improve diagnostic accuracy.

This meta-analysis is subject to certain limitations. For one, as all included studies were retrospective in design, these findings are subject to a high risk of bias. Moreover, many of the included studies failed to indicate whether patients were recruited consecutively, and this oversight may have influenced the diagnostic performance of the models developed in individual studies. Next, different models contained different predictive factors, and the diagnostic results were not only influenced by PET/CT, but also influenced by other factors. However, different models also have the similarity that the predictive models can provide the comprehensive and quanitative analysis for the PNs. Lastly, the included studies did not utilize consistent reference standards, again potentially impacting the resultant diagnostic accuracy.

## Conclusions

In summary, PET/CT-based models appear to exhibit promising diagnostic performance when used to distinguish between benign and malignant PNs, outperforming PET-derived SUV_max_ values alone when employed for the differential diagnosis of PNs.

## Data Availability

The data that support the findings of this study are available from the corresponding author upon reasonable request.

## Abbreviations

AUC: area under the curve
CEA: carcinoembryonic antigen
CT: computed tomography
FDG: fludeoxyglucose
FN: false negative
FP: true negative
NLR: negative likelihood ratio
PET: positron emission tomography
PLR: positive likelihood ratio
PN: pulmonary nodule
QUADAS-2: quality assessment of diagnostic accuracy studies
SROC: summary receiver operating characteristic
SUV_max_: standardized maximum uptake value
TN: true negative
TP: true positive
